# Rumen epithelial functional adaptation in alpine ewes: transcriptomic effects of micronutrient premix and concentrate supplementation levels

**DOI:** 10.1007/s44154-026-00324-2

**Published:** 2026-08-05

**Authors:** Jie Cheng, Yan Cheng, Baohui Zhang, Kefyalew Gebeyew, Aifei Yan, Fan Hu, Jinxia Li, Jie Liu, Weihua Zhao, Jinghe Kang, Zhiliang Tan, Zhixiong He

**Affiliations:** 1 State Key Laboratory of Forage Breeding-by-Design and Utilization, National Engineering Laboratory for Pollution Control and Waste Utilization in Livestock and Poultry Production, and Hunan Provincial Key Laboratory of Animal Nutritional Physiology and Metabolic Process, Institute of Subtropical Agriculture, Chinese Academy of Sciences, Changsha, Hunan 410125 China; 2 College of Advanced Agricultural Sciences, University of Chinese Academy of Sciences, Beijing, 100049 China; 3 Hulun Buir State Farm Hadatu Far, Hulun Buir, 021511 China; 4 Hulun Buir State Farm, Hulun Buir, 021008 China; 5 Yuelushan Laboratory, Changsha, Hunan 410128 China

**Keywords:** Alpine adaptation, Vitamin, Microelement, Rumen function, RNA-seq

## Abstract

Ovine species inhabiting harsh environments frequently develop enhanced functional adaptations. This study aims to (1) characterize rumen epithelial modifications in lactating Hulunbeier ewes under alpine conditions, and (2) elucidate how these adaptive responses are modulated by micronutrient supplementation and varying concentrate feed levels. Thirty lactating Hulunbeier ewes were randomly assigned to one of the three dietary groups and fed the same roughages with one of the following concentrates: 1) 550 g/d of local concentrate (LF) as the control, 2) 550 g/d of formulated concentrate (FF), and 3) 700 g/d of formulated concentrate (FS). Daily feed intake were recorded, and rumen epithelial tissues were collected for transcriptome analysis after slaughter on day 37 of the experiment. Transcriptomic analyses were performed on a subset of 11 ewes (LF, *n* = 4; FF, *n* = 4; FS, *n* = 3). No independent qPCR validation was conducted; results are therefore preliminary. In the exploratory cross-breed comparison, Hulunbeier sheep showed a suggestive trend toward enhanced expression of energy metabolism genes (ATP5MC2, ATP5PO, UQCRH) and genes encoding mitochondrial respiratory chain components relative to lowland Hu sheep. Supplementation with micronutrient premix upregulated key detoxification genes (CYP1A1, LOC101119706) and modulated immune-related pathways. In contrast, high concentrate levels promoted biosynthetic processes while suppressing cellular signaling functions. Gene Set Enrichment Analysis further indicated activation of thermogenesis pathways in the high-concentrate group, accompanied by altered expression of electron transport chain genes. PPARA may be involved in volatile fatty acid metabolism in response to micronutrient supplementation, but this requires direct experimental testing. Collectively, with the caveat of small sample size and lack of qPCR validation, these pilot-scale transcriptomic findings generate hypotheses that may underlie nutritional modulation of rumen function in alpine-adapted sheep; however, further validation with larger sample sizes and correlated phenotypic measurements is warranted. These results provide preliminary insights for developing targeted nutritional strategies to enhance ruminant production in breeds adapted to extreme environments.

**Supplementary Information:**

The online version contains supplementary material available at 10.1007/s44154-026-00324-2.

## Introduction

Environmental drivers elicit species-specific adaptations to multi-stress regimes—including high-altitude hypoxia, drought, and thermal extremes—via integrated genetic and phenotypic plasticity mechanisms in animal systems (Witt and Huerta-Sánchez 2019). Hulunbeier sheep are mainly raised on the Hulunbuir grassland, which experiences extremely cold and long winters (Zhang et al. 2019a). Typically, herders in Hulunbuir keep their sheep in barns during winter without heaters, and due to cost constraints, they only feed them hay and a small amount of simple concentrates. Meanwhile, breeding ewes under chilly conditions often experience a limitation of about 30% of their nutritional requirements (Sales et al. 2018), which causes them to require more nutritional intake for lactation. Therefore, we hypothesize that lactating Hulunbeier sheep developed environmentally adaptive traits modifiable through nutritional regulation.

The rumen, as a unique digestive organ of ruminants, has a variety of important physiological functions, including absorption, transportation, metabolic activity and host protection (Roh et al. 2007). Adaptive modifications in rumen physiological functions are usually recognized as the foundation of ruminant evolution (He et al. 2024). Volatile fatty acid (VFA) produced in the rumen can supply 70% of the energy requirements of ruminants (Flint et al. 2008). It has been shown that VFA is mainly absorbed by rumen epithelial cells through simple diffusion and bicarbonate-dependent protein-mediated transport (Aschenbach et al. 2009; López et al. 2003), and the absorbed VFA is then metabolized to ketone bodies in the basal and polyspinal layers of the rumen epithelium to produce energy (VI 1998). In addition, the rumen produces substantial amounts of endogenous heat during rumination and digestive metabolism, which can significantly influence the performance of ruminants (Ferreira et al. 2021). However, the mechanisms underlying the effects of different nutritional strategies on rumen function in lactating ewes under alpine conditions are still unknown.

Micronutrients are critically involved in the adaptive processes triggered by environmental stress (Rathod et al. 2025). Despite minimal required quantities, vitamins and trace elements are indispensable for sustaining physiological homeostasis, antioxidant defense, and immune competence (Lee 2004; Tous et al. 2014). Studies have shown that by modulating metabolic activity and enhancing antioxidant capacity, micronutrients enable animals to sustain energy provision and stabilize cellular function under extreme challenges including heat, cold, and starvation (Hardison and Eliason 2024; Velichko 2023). Specifically, micronutrients (e.g., by Zn, Se, Cu, Mn and vitamins E/C) confer cellular protection against extreme-environment-induced oxidative damage via free radical scavenging through activation of antioxidant enzymes (Velichko 2023). Meanwhile, differences in micronutrient intake can affect certain functions of the rumen (Kincaid 2000), including nutrient utilization efficiency (Perry and Hillier 1969), the total VFA and individual butyrate production (Pino and Heinrichs 2016). While herbage contains some micronutrients, it is unable to satisfy the needs of Hulunbeier sheep. This is especially true in the current intensive production system, where these impacts are amplified and directly affect the animal's health and the farm's profitability.

Previous research has demonstrated that both pelleted concentrate supplementation and environmental variations can alter the transcriptomic profile of the ovine rumen (Xue et al. 2021; Zhang et al. 2016). To build upon this foundation, the present study employed RNA-seq to (1) characterize the adaptive rumen transcriptome of Hulunbeier sheep in an alpine environment, and (2) decipher nutrient-driven regulatory mechanisms governing rumen adaptation under these extreme conditions. However, direct cross-breed transcriptomic comparisons remain confounded by study-specific conditions. Our exploratory comparison described below is hypothesis-generating only.

## Results

### Transcriptome profiles of rumen epithelium

Thirty healthy lactating Hulunbeier ewes, with an average initial body weight of 55.50 ± 1.04 kg, were evenly and randomly divided into three groups and housed in three individual pens. The sheep in each pen randomly obtained one of the following diets. 1) 550 g/d of local concentrate and 2000 g/d of rapeseed straw (LF) for one sheep in one day as the control, 2) 550 g/d of formulated concentrate and 2000 g/d of rapeseed straw for one sheep in one day (FF), and 3) 700 g/d of formulated concentrate and 2000 g/d of rapeseed straw for one sheep in one day (FS). On day 37 of the experiment, before morning feeding, four sheep were randomly selected from the LF and FF groups, and three sheep were randomly selected from the FS group for bloodletting slaughter. Samples of rumen epithelium were collected from the same position at the top of the rumen within 10 min of slaughter. After RNA sequencing of 11 rumen epithelium samples, a total of 281.58 million clean reads were obtained, with an average of 25.6 ± 1.41 million reads per sample. About 26.07, 26.07, and 24.34 million valid reads were detected from individual samples of LF, FF, and FS groups, respectively. The clean reads were aligned to the sequence of the sheep genome (Ovis aries, reference genome version: GCF_002742125.1_Oar_rambouillet_v1.0). The average mapping rates for LF, FF, and FS groups were 71.73 ± 1.44%, 72.42 ± 1.46%, and 67.71 ± 2.44%, respectively. The unique mapping rate of reads for LF, FF, and FS groups were 56.55 ± 0.83%, 58.26 ± 0.83%, and 56.55 ± 0.83%, respectively (Supplementary Table S1). After data processing, approximately 11,577, 11,931, and 11,109 expressed genes (genes with an average FPKM > 1 for each sample) were detected in rumen epithelial tissues of LF, FF, and FS groups, respectively.

### Exploratory comparison of rumen transcriptomes between Hulunbeier and Hu sheep

**Fig. 1:**
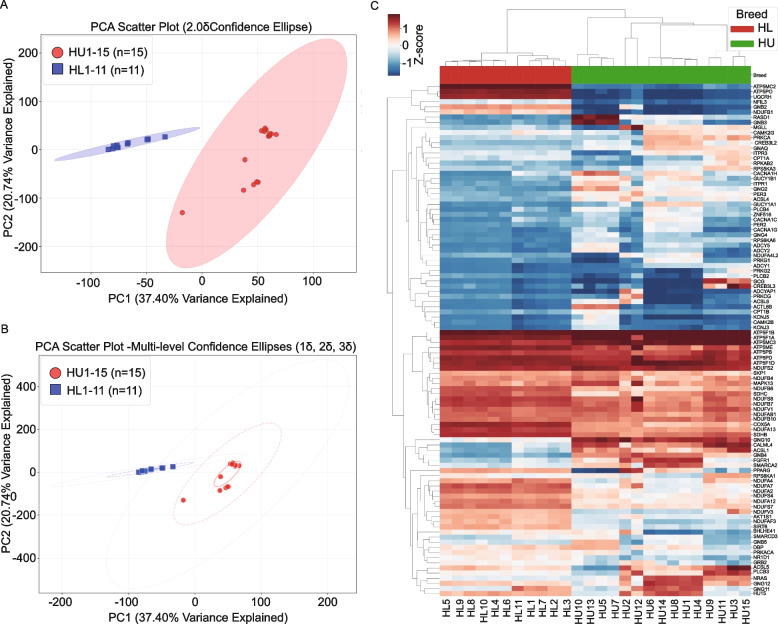
Comparison of rumen transcriptomic profiles between Hulunbeier sheep (HL) and Hu sheep (HU). **A** PCA of rumen transcriptomes from Hulunbeier sheep and Hu sheep (2.0σ confidence ellipses). **B** PCA of rumen transcriptomes from Hulunbeier sheep and Hu sheep (multi‑level confidence ellipses). **C** Clustering heatmap of the top 100 genes differing most in environmentally adaptive pathways between HL and HU groups. Note: This comparison is exploratory and confounded by study-specific conditions. Interpret with caution; not indicative of breed-specific effects alone

After data processing, 9,698 genes were identified as commonly and robustly expressed across all samples from both Hulunbeier (HL) and Hu sheep (HU). PCA revealed a clear segregation between the two breeds, with the first two principal components (PC1: 37.40%; PC2: 20.74%) accounting for a cumulative 58.14% of the total transcriptomic variance (Fig. 1A). This distinct clustering were further supported by 2.0σ confidence ellipses, underscoring significant divergence in global transcriptomic profiles between the two breeds (Fig. 1B). Heatmap clustering analysis unveiled pronounced breed-specific expression patterns (Fig. 1C). Genes upregulated in HL sheep were significantly enriched in functions related to energy metabolism (ATP5MC2, ATP5PO, UQCRH) and mitochondrial respiratory chain function (NDUFA2, NDUFA7). Conversely, HU-upregulated genes were predominantly associated with lipid metabolism and signal transduction (CALML4, ACSL1, GNB4). However, because the Hu sheep data originated from a different study, this comparison is exploratory and cannot distinguish breed-specific from study-specific effects.

### Effect of micronutrient premix on rumen epithelial transcriptome

**Fig. 2:**
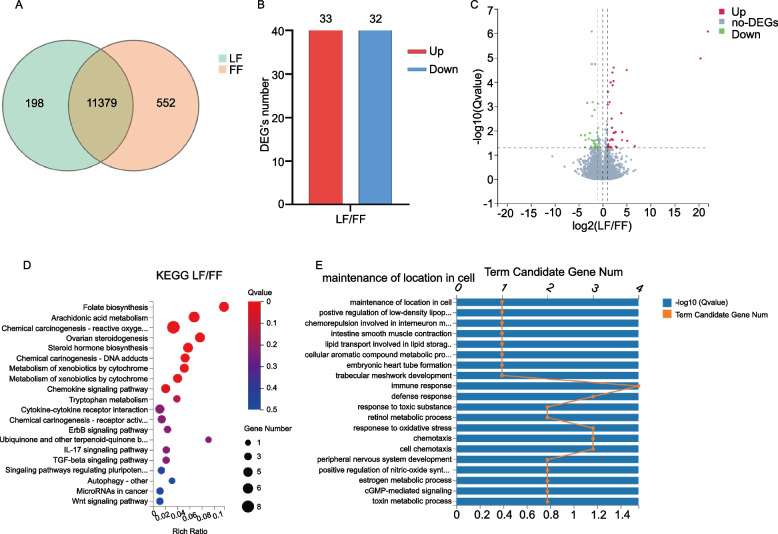
Intergroup differential gene and enrichment pathway analysis of LF/FF. **a** Number of unique and shared expressed genes in LF/FF. **b** Distribution statistics of differential expression genes (DEGs) in LF/FF. **c** Volcano plot distribution of DEGs in LF/FF. **d**-**e** Results of KEGG and GO biological process pathway enrichment of DEGs in LF/FF

Further analysis of the expressed genes showed that 11,379 genes were shared between the LF and FF groups, with 198 and 552 genes unique to the LF and FF groups, respectively (Fig. 2A). Among the shared genes, 65 genes were differentially expressed between FF and LF groups (|log2FC|> = 1; Q-value < = 0.05), of which 32 were upregulated and 33 were downregulated in the FF group (Fig. 2B). The volcano plot shows a clear picture of their distribution (Fig. 2C). KEGG pathway analysis showed that the 65 differentially expressed genes (DEGs) between the LF and FF groups were mainly enriched in pathways such as folate biosynthesis, arachidonic acid metabolism, ovarian steroidogenesis, steroid hormone biosynthesis, metabolism of xenobiotics by cytochrome, viral protein interaction with cytokine, tryptophan metabolism, cytokine-cytokine receptor interaction and so on (Fig. 2D). Specifically, the upregulated DEGs were mainly involved in arachidonic acid metabolism, folate biosynthesis, viral protein interaction with cytokine, ovarian steroidogenesis, steroid hormone biosynthesis and other pathways (Supplementary Fig. S1a). Similarly, the down-regulated DEGs were primarily involved in tryptophan metabolism, ovarian steroidogenesis, metabolism of xenobiotics by cytochrome, steroid hormone biosynthesis, and other pathways (Supplementary Fig. S1b). Functional classification results showed that DEGs were mainly enriched in Gene Ontology (GO) terms related to biological processes such as immune response, defense response, response to oxidative stress, chemotaxis, cell chemotaxis, response to toxic substance, retinol response to toxic substance, retinol metabolic process and other terms (Fig. 2E).

### Effect of concentrate supplementary level on rumen epithelial transcriptome

**Fig. 3:**
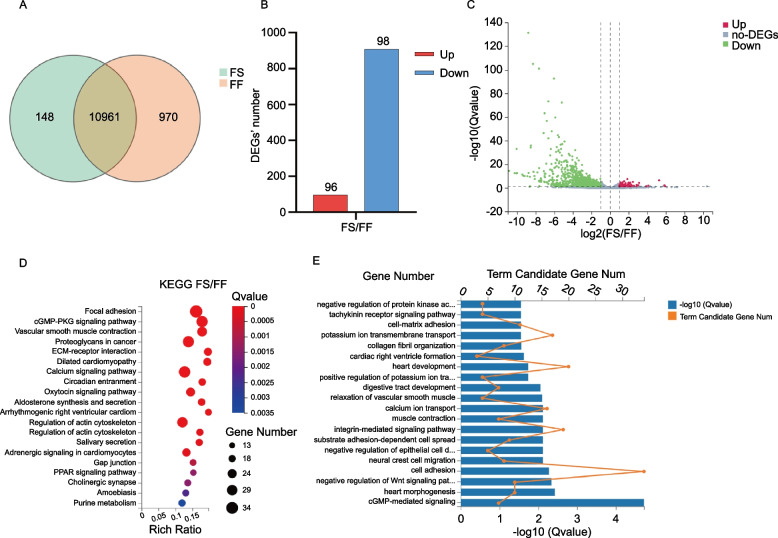
Intergroup differential gene and enrichment pathway analysis of FF/FS. **A** Number of unique and shared expressed genes in FF/FS. **B** Distribution statistics of differential expression genes (DEGs) in FS/FF. **C** Volcano plot distribution of DEGs in FF/FS. **D**-**E** Results of KEGG and GO biological process pathway enrichment of DEGs in FF/FS

Similarly, 10,961 shared expressed genes were found in the FS and FF groups, with 148 and 970 genes specifically expressed in the FS and FF groups, respectively (Fig. 3A). A total of 1004 DEGs were found between FS and FF groups, among which 96 genes were upregulated and 908 genes were downregulated in the FS group (Fig. 3B). The volcano diagram showed a better distribution (Fig. 3C). The KEGG pathway analysis revealed that DEGs in FS/FF were mainly enriched in pathways like focal adhesion, cGMP-PKG signaling pathway, vascular smooth muscle contraction, proteoglycans in cancer, calcium signaling pathway, oxytocin signaling pathway, oxytocin signaling pathway, regulation of actin cytoskeleton and adrenergic signaling in cardiomyocytes (Fig. 3D). Specifically, the upregulated DEGs were mainly involved in cGMP-PKG signaling pathway, focal adhesion, vascular smooth muscle contraction, dilated cardiomyopathy (DCM), calcium signaling pathway, ECM-receptor interaction, oxytocin signaling pathway, regulation of actin cytoskeleton, salivary secretion and circadian entrainment (Supplementary Fig. S1c). The down-regulated DEGs were mainly involved in arachidonic acid metabolism, steroid hormone biosynthesis, biosynthesis of amino acids, and other pathways (Supplementary Fig. S1d). Functional categorization results showed that DEGs were mainly enriched in the GO terms for biological processes such as cell adhesion, heart development, potassium ion transmembrane transport, integrin-mediated signaling pathway, cell–matrix adhesion, and calcium ion transport (Fig. 3E).

### Increased dietary concentrate levels activate thermogenesis pathways in the rumen epithelium of sheep

**Fig. 4:**
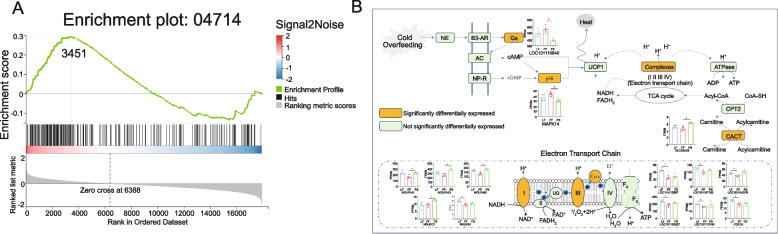
Effects of dietary intervention on rumen thermogenic performance in Hulunbeier sheep. **A** GSEA enrichment plot for the thermogenesis pathway (KEGG 04714) in FS vs. FF (NES = 1.57, FDR q = 0.027). **B** Effect of dietary intervention on gene expression within the thermogenesis pathway

Rumen thermogenesis is a critical mechanism for cold adaptation in ruminants, including lactating ewes. GSEA demonstrated significant upregulation of thermogenesis-related pathways in the FS group compared to the FF group (Fig. 4A, Normalized Enrichment Score [NES] = 1.57, FDR q-value = 0.027). The enrichment plot for the thermogenesis pathway (KEGG 04714) showed a clear positive enrichment, with a peak at the 6388th rank in the gene list ordered by signal-to-noise ratio. The leading-edge subset analysis further identified core genes contributing to this enrichment signal. These results collectively indicate coordinated activation of thermogenic pathways in the FS group, suggesting enhanced ruminal thermogenic capacity. To further elucidate the molecular basis underlying this activation, we retrieved 285 genes annotated to the KEGG thermogenesis pathway (map04714). Cross-referencing this gene set with our rumen transcriptomic data identified 14 genes that were differentially expressed between the FS and FF groups (Fig. 4B). These differentially expressed genes encode key components of the thermogenesis pathway, including Gs protein (LOC101115640), p38 MAPK (MAPK14), CACT (SLC25A29), and the electron transport chain subunits, implying that dietary concentrate-induced alterations impact ruminal thermogenic function.

### Effect of micronutrient premix addition on gene expression related to rumen epithelial immunity and VFA utilization

**Fig. 5:**
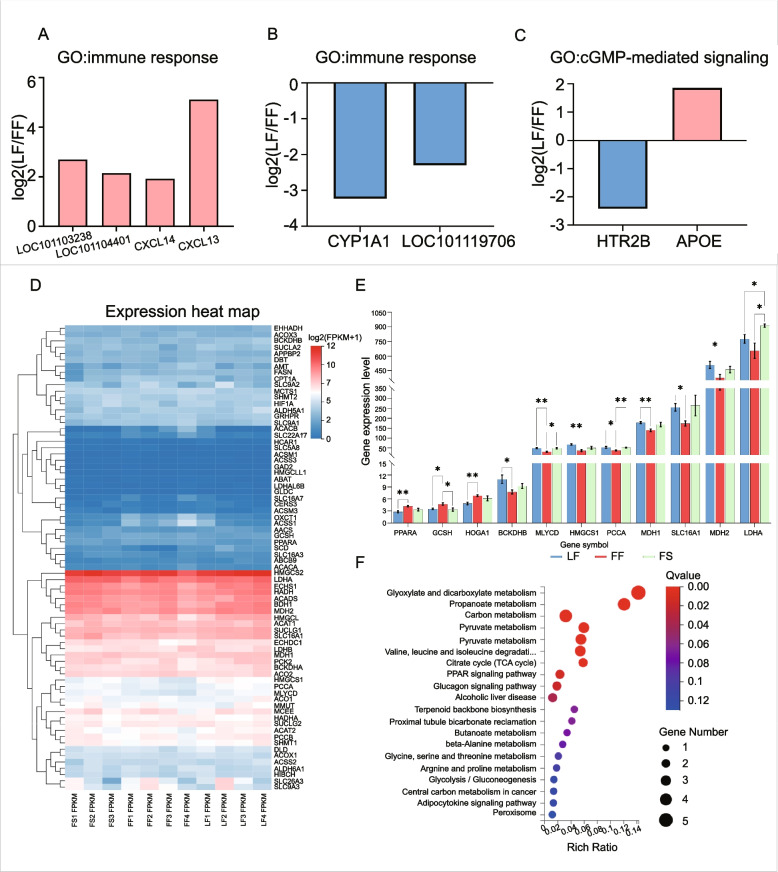
Analysis of genes related to immunity and volatile fatty acids (VFA) utilization in the rumen. **A**-**C** Expression of DEGs enriched in immune-related GO terms. **D** Heatmap of VFA utilization-associated genes. **E** Differential expression of VFA utilization-associated genes (* represents *P* < 0.05, ** represents *P* < 0.01, and *** represents *P* < 0.001). **F** KEGG pathway enrichment results for DEGs associated with VFA utilization

To assess the effect of micronutrient premix addition on the expression of genes related to rumen epithelial immunity and VFA utilization, we further analyzed the enrichment results. Among the GO terms for DEGs in the FF/LF, four DEGs (LOC101103238, LOC101104401, CXCL14, CXCL13) (Fig. 5A) were up-regulated, and two DEGs (CYP1A1, LOC101119706) were down-regulated (Fig. 5B) within the immunity-related GO terms (immune response and response to toxic substance). In the signaling-regulation-related GO term (cGMP-mediated signaling), the expressions of APOE and HTR2B were up-and down-regulated, respectively (Fig. 5C). In addition, using data from the KEGG official website and other literature, we mapped the heat map of genes related to VFA utilization based on FPKM values (Fig. 5D). The results of the differential analysis showed that the expression of SLC16A1, which is involved in VFA uptake and transport, was significantly higher in the LF than in the FF group. The expression of PPARA, GCSH, HOGA1, BCKDHB, MLYCD, HMGCS1, PCCA and MHD2, which are involved in VFA metabolism, showed significant differences among the groups (Fig. 5E). Enrichment of these DEGs for KEGG pathway revealed that PCCA, BCKDHB and MLYCD were enriched in Propanoate metabolism and HMGCS1 was enriched in Butanoate metabolism (Fig. 5F). Meanwhile, a protein–protein interaction and co-expression network analysis (performed with the BGI Dr. Tom platform, default settings) predicted associations between HMGCS1 and PPARA, as well as MDH1 and MDH2 (Supplementary Fig. S2). These are computational predictions, not experimentally validated.

## Discussion

Previous research has demonstrated that nutritional management strategies, such as precision feeding and customized total mixed rations, effectively improve milk production and metabolic status in ewes. Such strategies help maintain body condition and support resilience to environmental stresses, which is critical for sustaining productivity under challenging conditions (Barrio et al. 2025). Furthermore, specific dietary modifications, such as micronutrient supplementation and graded concentrate levels, are known to modulate rumen physiological dynamics in ruminants, as evidenced by recent meta-analyses (Vigh et al. 2023). However, the molecular responses to these nutritional interventions in lactating Hulunbeier ewes remain uncharacterized. To address this knowledge gap, we employed transcriptomic analysis to characterize the molecular response of the rumen epithelium in lactating Hulunbeier sheep to dietary regimens under alpine conditions. While Hulunbeier sheep are adapted to alpine environments, the trial was conducted under mild conditions (average 11.5 °C), indicating that observed differences primarily reflect breed background rather than acute cold stress.

### Transcriptome characterization of lactating Hulunbeier ewes’ rumen epithelium

Harsh alpine environments impose substantial physiological and metabolic challenges on sheep. Previous studies have shown that DEGs in the rumen epithelium during cold seasons are predominantly enriched in pathways such as PPAR signaling, phagosome formation, Legionellosis, arginine and proline metabolism, and cytochrome P450–mediated xenobiotic metabolism. These pathways, which govern lipid metabolism, energy regulation, immune response, and detoxification, constitute a molecular basis for cold adaptation (Liu et al. 2022). In this exploratory comparison, PCA revealed a separation between the two breeds, suggestive of potential differences in global gene expression profiles, though this may also reflect study-specific conditions. This divergence was further reflected in heatmap visualizations as breed‑specific expression modules: Hulunbeier sheep exhibited marked enrichment in energy metabolism–related genes (ATP5MC2, ATP5PO, UQCRH) and mitochondrial respiratory chain components (NDUFA2, NDUFA7), whereas Hu sheep showed preferential expression of genes involved in lipid metabolism and signal transduction (CALML4, ACSL1). This expression pattern shows parallels with adaptive strategies reported in other high-altitude ruminants, whereby enhanced energy metabolic efficiency and mitochondrial function support the elevated thermogenic demands associated with cold exposure (Sha et al. 2023; Sha et al. 2022). These findings underscore the importance of developing targeted nutritional interventions to enhance these innate adaptive mechanisms through precision modulation of nutrient utilization efficiency and stress resilience pathways. However, a key limitation of this comparative analysis is that the Hu sheep transcriptomic data were generated from different experimental conditions and sequencing platforms. Therefore, these breed-specific conclusions are preliminary and should be validated in future studies under controlled, parallel conditions.

### Effects of micronutrient premix on the physiological function of rumen epithelium

A comparison of the FF and LF groups identified 65 DEGs. The up‑regulated DEGs were primarily enriched in steroid biosynthesis and signal transduction pathways, such as CYP1A1 and LOC101119706. These genes encode members of the cytochrome P450 protein family, which are key players in xenobiotic metabolism and inflammation mitigation processes (Mariz et al. 2018). This upregulation is consistent with a hypothesis that the formulated concentrate may influence detoxification pathways in the rumen epithelium, a finding consistent with previous multi-omics analysis (Liu et al. 2022). In addition, the up-regulation of the PPAR signaling pathway and other related signaling pathways in the FF group not only further demonstrated the beneficial effects of formulated concentrates in alleviating intestinal inflammation (Daynes and Jones 2002), but also revealed their function in promoting fat metabolism (Yuan 2015). Conversely, the downregulation of immune-related chemokine genes, such as CXCL13 and CXCL14, may reflect a response to the dietary shift. The increased concentrate level can elevate ruminal volatile fatty acid (VFA) and toxin concentrations, potentially suppressing certain immune signals, as previously observed in cattle (Gholizadeh et al. 2020). Specifically, the expression of CYP1A1 and LOC101119706, genes involved in response to toxic substances, was relatively higher in the FF group than in the LF group. These genes are known to metabolism of ruminal toxins, such as ethanol and other xenobiotics (Zhao et al. 2017). This suggests that micronutrient supplementation may upregulate detoxification genes potentially involved in ruminal toxin metabolism, a mechanism supported by previous findings (Maikanov et al. 2020). We also observed opposing regulatory effects. Notably, upregulation of HTR2B in the cGMP signaling pathway may confer protective effects by inhibiting lipid peroxidation (Tu et al. 2023). In contrast, down‑regulation of APOE in the FF group could impair the regulation of lipoprotein metabolism and cholesterol homeostasis (Fuentes et al. 2018). The downregulation of APOE may reflect a metabolic priority shift under dietary intervention. Overall, these results generate the hypothesis that micronutrient premix supplementation may influence immune and metabolic gene expression in the rumen epithelium. However, the net physiological impact must be further evaluated with phenotypic data, including direct measurements of inflammatory cytokines and ruminal toxin concentrations.

### Complex effects of increased concentrate level on rumen function and thermogenic pathways

Increasing the concentrate supplementation level within a certain range can enhance the digestibility of nutrients (Liu et al. 2005). However, excess concentrate can produce acid after fermentation in the rumen, leading to a decrease in pH and resulting in inflammation and acidosis (Giger-Reverdin et al. 2014). The probable cause of this result is a high level of concentrated diets, which disrupts the balance between apoptosis and proliferation of rumen epithelial cells and impairs epithelial cell integrity (Lu et al. 2018), untimely leading to a lesion. In the present study, the feed intake of ewes in the FS group was significantly higher than that in the FF group (Supplementary Fig. S3). This difference in nutrient load was associated with the identification of 1004 DEGs between the two groups. Upregulated genes were significantly enriched in pathways of folate, steroid hormone, and amino acid biosynthesis, which is consistent with a hypothesis of promoted anabolic metabolism in response to the higher concentrate intake. In contrast, downregulated genes were markedly enriched in key cellular regulatory pathways, such as the cGMP‑PKG signaling pathway, calcium signaling pathway, and focal adhesion, suggesting a hypothesis that a high dietary concentrate level could be associated with reduced efficiency of cellular signal transduction within the rumen epithelium. These findings are consistent with previous reports that high concentrate intake leads to rumen epithelial dysfunction (Lu et al. 2018).

### Analysis of differentially expressed genes and pathways related to thermogenesis and VFA utilization

Hulunbeier sheep live in high-altitude and cold environments, and supplemental concentrates can effectively help them resist the cold and maintain a high level of performance (Zhang et al. 2022). Meanwhile, VFAs absorbed and utilized through the rumen wall are the main energy substrate for ruminants (Flint et al. 2008). Therefore, elucidating the molecular mechanism by which micronutrient-enriched concentrates affect thermogenesis through altering VFA uptake and utilization in the rumen is an important guide for sheep rearing in alpine areas. Oxidative phosphorylation in mitochondria is the primary means by which animals generate ATP to sustain life. GSEA revealed a significant upregulation of thermogenic pathways (map04714) in the FS group, pointing to a major shift in energy metabolism. Paradoxically, within this activated pathway, genes encoding Gs (LOC101115640) and p38 (MAPK14) were downregulated in the FS group. This implies a constraint on protein kinase A (PKA) activation, which would reduce the transcriptional stimulation of UCP1 and potentially diminish one arm of thermogenic performance (Cao et al. 2004; Zhang, et al. 2019b). In contrast, the FS group showed upregulation of genes encoding CACT (SLC25A29) and mitochondrial Complex I subunits (NDUFA11, NDUFA13, NDUFAB1, NDUFC1). This upregulation would facilitate fatty acid transport and oxidation, promoting substrate-driven energy (heat) production (Stanca et al. 2024), while enhanced electron transport chain (ETC) activity increases the proton gradient that indirectly drives uncoupling thermogenesis via UCP1, thereby improving overall thermogenic efficiency (Tabuchi and Sul 2021). We hypothesize that FS compensates for the impaired Gs/p38–UCP1 axis by co-opting an alternative thermogenic strategy via upregulating fatty acid oxidation pathways (CACT and Complex I). This reflects a state of metabolic flexibility under conditions of “energy surplus” or “cold stress,” where fatty acids are channeled toward heat production and ETC activity is optimized for both ATP synthesis and heat generation. Conversely, the broad downregulation of ETC-related genes in FF (including genes affecting Complex I, III, and cytochrome c assembly) suggests a state of metabolic conservation under “energy deficit” or “normothermic homeostasis,” where non-essential energy expenditure like thermogenesis is suppressed to prioritize basal ATP production. These findings provide novel molecular insights into metabolic adaptability in the rumen, with potential implications for nutritional strategies aimed at improving energy homeostasis, although further experimental validation is required.

It has been demonstrated that micronutrient intake levels affect the efficiency of VFA utilization in the rumen epithelium. For example, group B vitamins can enhance VFA production and increase ammonia and nitrogen utilization in the rumen (Parnian-Khajehdizaj et al. 2018), while Se promotes intra-ruminal fermentation and influences the metabolism of propionic acid (Wei et al. 2019). In the present study, the FF group showed significantly lower expression of key genes involved in VFA metabolism, including PCCA, BCKDHB, and MLYCD in propanoate metabolism and HMGCS1 in butanoate metabolism, compared to the LF group. The KEGG enrichment analysis generates the hypothesis that micronutrient supplementation could influence the PPARA signaling pathway (Li et al. 2021), potentially correlating with altered expression of VFA metabolism genes, thereby reducing the relative utilization efficiency of VFAs by the rumen epithelium. Computational predictions suggest potential PPARA-HMGCS1 associations (Supplementary Fig. S2), though this requires experimental validation. This is consistent with previous reports of PPARA's role in suppressing VFA metabolic genes (Neubauer et al. 2019). Conversely, in the LF group, a relative deficiency in micronutrients like vitamin A may decrease malonyl‑CoA synthesis, thereby disinhibiting fatty acid oxidation (Oliveros et al. 2007). This shift in metabolic fuel preference may indirectly enhance the metabolic efficiency of VFAs by reducing competition for mitochondrial cofactors. These results suggest an antagonistic interaction between micronutrients and concentrate intakes. Thus, future studies employing multi‑gradient experimental designs are needed to clarify this dose‑dependent relationship.

## Conclusion

This pilot study (*n* = 4,4,3; no qPCR validation) generates the hypothesis that the rumen epithelium of Hulunbeier sheep exhibits transcriptomic characteristics potentially relevant to high-altitude cold adaptation. We found exploratory evidence that a micronutrient premix may influence genes involved in detoxification, lipid metabolism, and immunity, while a higher concentrate level was associated with anabolic biosynthesis and suppressed cellular signaling pathways. These correlational findings are hypothesis-generating only and are constrained by small sample size, lack of orthogonal validation, and batch effects in cross-breed comparisons. Future studies with larger cohorts, parallel breed comparisons, qPCR validation, and functional assays are required.

## Materials and methods

### Experimental diet

The experimental diets were formulated by combining the feeding practices of local herders with the current feeding standards in China (NY/T 816–2021), ensuring they fully met the nutritional requirements of lactating Hulunbeier ewes. The main difference between formulated concentrate and local concentrate is the addition of micronutrient premix in formulated concentrate, and the specific composition and nutritional components are shown in Table 1. The concentrate used was made in the local processing plant according to the formula, and the rapeseed straw was purchased from local herders.

**Table 1:** Dietary formula for experimental sheep

Item	Formula concentrate	Local concentrate	Forage
Ingredients, %			
Corn	57.86	30.00	0.00
Barley	13.46	30.00	0.00
Wheat bran	9.20	0.00	0.00
Soybean meal	3.56	0.00	0.00
Cottonseed meal	8.13	0.00	0.00
Premix1	5.25	0.00	0.00
Beet molasses	2.54	0.00	0.00
Rape meal	0.00	20.00	0.00
Wheat	0.00	20.00	0.00
Rapeseed straw	0.00	0.00	100.00
Total	100.00	100.00	100.00
Nutritional level			
DM (%)	93.06	92.74	95.11
ASH (%)	8.03	5.53	8.02
EE (%)	9.78	12.59	9.05
NDF (%)	14.36	22.05	52.91
ADF (%)	4.96	9.06	38.56
CP (%)	17.78	16.30	11.16
Ca (%)	1.48	0.57	1.47
P (%)	0.68	0.45	0.29
GE (MJ/Kg)	15.98	17.63	16.84

All data presented in the table are measured values obtained as per the Chinese National Standard GB/T 14924.9–2001, Laboratory Animals—Formula Feeds—Determination of Routine Nutrients. The premix provides per kg of feed: iron 29.45 mg, copper 7.36 mg, zinc 38.65 mg, manganese 26.69 mg, iodine 0.74 mg, cobalt 0.15 mg, selenium 0.06 mg, vitamin A 2,310 IU, vitamin D 280 IU, and vitamin E 23 IU

### Animals and experiment design

The animal trials were conducted from May to June on a private livestock farm in Hadatu Town, Hulunbuir. According to meteorological data from the China Meteorological Administration, the local climate during this experimental period was characterized by low temperatures (average: 11.5 °C), moderate humidity (average: 46%), and substantial sunshine duration (average: 9.2 h per day). Observed transcriptomic patterns therefore reflect breed background and nutritional interventions, not acute cold exposure. Thirty healthy lactating Hulunbeier ewes (Red-headed short-tailed sheep), with an average initial body weight of 55.50 ± 1.04 kg, were evenly and randomly (by using the Rand function in Excel) divided into three groups and housed in three individual pens. The sheep in each pen randomly obtained one of the following diets. 1) 550 g/d of local concentrate and 2000 g/d of rapeseed straw (LF) for one sheep in one day as the control, 2) 550 g/d of formulated concentrate and 2000 g/d of rapeseed straw for one sheep in one day (FF), and 3) 700 g/d of formulated concentrate and 2000 g/d of rapeseed straw for one sheep in one day (FS). The experiment lasted for a total of 46 days, including a 10-day pre-experimental period and a 36-day formal experimental period. Prior to the experiment, the pens were disinfected, and all sheep were dewormed and vaccinated. The sheep were fed by herd at 9 a.m. and 4 p.m. every day following the dietary standard, and the leftovers from the previous day were weighed and recorded before morning feeding. All sheep were given free access to water.

### Sample collection

On day 37 of the experiment, before morning feeding, four sheep were randomly selected from the LF and FF groups, and three sheep were randomly selected from the FS group for bloodletting slaughter. Samples of rumen epithelium were collected from the same position at the top of the rumen within 10 min of slaughter. These samples were placed in sterile, enzyme-free sample bags, immediately snap frozen in liquid nitrogen, and then transferred to a refrigerator at −80℃ for RNA sequencing.

### RNA extraction and sequencing of rumen tissue

Total RNA was extracted from rumen samples using the TRIzol kit (Invitrogen, CA, USA) according to the manufacturer's instructions. Total RNA was then processed for the construction of cDNA libraries using the mRNA enrichment method. Briefly, mRNA with polyA tails was enriched using magnetic beads with Oligo (dT) (Invitrogen, CA, USA). The obtained RNA was fragmented with an interrupting buffer, and a random N6 primer was used for reverse transcription to synthesize a single-stranded cDNA, and then the cDNA duplex was synthesized to form a double-stranded DNA. The synthesized double-stranded DNA was flattened out and phosphorylated at the 5' end, and then the 3' end was formed to form a sticky end protruding from the "A", and then a double-stranded DNA was ligated to form a double-stranded DNA with a protruding "T" at the 3' end. A bulge-like junction with a protruding "T" at the 3' end is then attached. The ligated product is amplified by PCR using specific primers; the PCR product is heat denatured to a single strand, and the single-stranded DNA is looped with a bridge primer to obtain a single-stranded circular DNA library. Then, RNA-seq was performed using the BGISEQ-500 platform to obtain raw data.

### Transcriptome mapping

The raw data obtained from sequencing were filtered using SOAPnuke (v1.5.6) to filter out 1) reads containing junctions (junction contamination), 2) reads with unknown base N content greater than 5%, and 3) low-quality reads (reads with bases of quality value less than 15 accounting for more than 20% of the total number of bases in the read are considered as Low-quality reads), thereby obtaining clean data. The clean data were then aligned to the reference genome using HISAT2 (v2.1.0) software. Clean data was compared to the reference gene set using Bowtie2 (v2.3.4.3). Gene expression was quantified using RSEM (v1.3.1) software, and a clustered heatmap of gene expression across different samples was plotted using pheatmap (v1.0.8) (Raivo Kolde. Package ‘pheatmap’. 2019–01–04 13:50:12 UTC). Differentially expressed genes were detected using DESeq2 (v1.4.5) with a Q-value ≤ 0.05. Gene Set Enrichment Analysis (GSEA) was conducted using the Dr. Tom cloud platform (BGI-Genomics).

###  Geneontology and KEGG enrichment analysis of DEGs

To further explore the gene functions associated with phenotypic changes, we performed Gene Ontology (GO) (http://www.geneontology.org) and Kyoto Encyclopedia of Genes and Genomes (KEGG) (https://www.kegg.jp) enrichment analysis of differentially expressed genes (DEGs) based on the hypergeometric test using the ‘phyper’ function (https://en.wikipedia.org/wiki/Hypergeometric_distribution) in the R software, and calculated the P value. The P-value was then adjusted for FDR to obtain the Q value. The Q-value was used to identify the KEGG pathways and GO terms, and the function with a Q-value < = 0.05 was usually considered as significantly enriched.

###  Comparison of rumen transcriptome characteristics between Hulunbeier and Hu sheep

Environmental adaptation in sheep breeds is driven by transcriptional regulation divergence across ecological gradients (Yang et al. 2016). Comparative transcriptomic analysis of rumen epithelium between cold-adapted Hulunbeier sheep and lowland Hu sheep is able to reveal functional adaptations to different environmental stressors. Based on the raw sequencing data of rumen transcriptomes from 15 Hu sheep obtained from previous studies (Xue et al. 2021), we processed the data through uniform cleaning, quality control, and alignment procedures to generate standardized FPKM values. We then filtered out low-expression genes (mean FPKM < 1) and applied a log2 transformation to the remaining expression matrix. Principal component analysis (PCA) was performed to assess global transcriptomic variations and sample relationships. For a targeted analysis, we retrieved the comprehensive gene list for ovine Environmental Adaptation pathways from the KEGG database (entry br08901) and generated a heatmap visualizing the top 100 differentially expressed genes between Hulunbeier and Hu sheep within these pathways.

###  Statistical analysis

A one-way analysis of variance (ANOVA) with Bonferroni correction for multiple testing was applied to genes within the thermogenesis pathway using Python. The significance threshold was adjusted to α = 0.05/14 ≈ 0.0036. Corrected *p*-values < 0.0036 were considered statistically significant. All bar graphs were generated and visualized using GraphPad Prism 9.0. Data are expressed as mean ± SEM, and differences with a *P*-value < 0.05 were considered statistically significant.

## Supplementary Information

Supplementary Material 1.

## Data Availability

The datasets generated and analysed during the current study are available in the Sequence Read Archive (SRA) database, https://www.ncbi.nlm.nih.gov/search/all/?term=PRJNA1099538 and Science Data Bank (Science DB), https://www.scidb.cn/en/anonymous/UUIzMlVu.

## Abbreviations

VFA: Volatile fatty acid
DEGs: Differentially expressed genes
GO: Gene Ontology
KEGG: Kyoto Encyclopedia of Genes and Genomes
GSEA: Gene Set Enrichment Analysis
NES: Normalized Enrichment Score
PCA: Principal component analysis
COX: Cytochrome c oxidase
ETC: Electron transport chain
