# Supplementary material for: Rumen epithelial functional adaptation in alpine ewes: transcriptomic effects of micronutrient premix and concentrate supplementation levels
Source: Stress Biol. 2026 Aug 5;6(1):56. doi: 10.1007/s44154-026-00324-2 (PMC13442766; doi:10.1007/s44154-026-00324-2)
Supplement: Supplementary file 1 [file 44154_2026_324_MOESM1_ESM.docx]

**Supplementary Table S1**

Transcriptome sequencing results of rumen epithelium in each group.

| Statistics term | LF | FF | FS |
| --- | --- | --- | --- |
| Clean Reads (M) | 26.07±0.02 | 26.07±0.01 | 24.34±2.44 |
| Mapping Rate (%) | 71.73±1.44 | 72.42±1.46 | 67.71±2.44 |
| Uniquely Mapping Rate (%) | 56.55±0.83 | 58.26±1.60 | 52.80±1.88 |

**Supplementary Fig. S1**

KEGG enrichment results for DEGs in LF/FF and FS/FF. a KEGG enrichment results for upregulated DEGs in LF/FF. b KEGG enrichment results for downregulated DEGs in LF/FF. c KEGG enrichment results for upregulated DEGs in FS/FF. d KEGG enrichment results for downregulated DEGs in FS/FF.

**Supplementary Fig. S2**

Linkage map of VFA utilization-associated DEGs.

**Supplementary Fig. S3**

Feed intake statistics during the experimental period.
